# Effects of exercise training on cardiotoxicity in cancer survivors. A systematic review

**DOI:** 10.12688/f1000research.112667.1

**Published:** 2022-05-05

**Authors:** Ravindra Reddy C, Stephen Samuel, Vijay Pratap Singh, Sourjya Banerjee

**Affiliations:** 1 Department of Physiotherapy, Kasturba Medical College, Mangalore, Manipal Academy of Higher Education, Manipal, India; 2 Department of Radiotherapy and Oncology, Kasturba Medical College, Mangalore, Manipal Academy of Higher Education, Manipal, India

**Keywords:** Exercise, Cardiotoxicity, Cancer Survivors, Rehabilitation

## Abstract

Background:

Cardiotoxicity is a major long-term complication of anti-cancer drugs such as anthracycline and androgen deprivation therapy (ADT). These drugs also impact the quality of life, reduced functional capacity, and life expectancy. Exercise attenuates the cardiotoxic effects of anticancer treatments, as indicated by a growing body of evidence.

Methods:

Studies for this review were retrieved from databases PubMed, SCOPUS, EMBASE, COCHRANE, and Web of Science and were restricted only to clinical trials. Study results were screened and synchronized to Mendeley. Studies that met the eligibility criteria were extracted into the spreadsheet, summarizing information regarding the site and cancer stages, adjuvant therapy, various exercise interventions, and outcome measures. Risk of bias quality analysis was done in accordance with the National Heart Lung Blood Institute.

Results:

In this systematic review, 9021 articles were screened. After the exclusion criteria, seven articles were included for qualitative analysis. Outcome measures analyzed were measures of cardiotoxicity such as left ventricular ejection fraction (LVEF), cardiac biomarkers, and global longitudinal strain.

Conclusion:

Although a structured exercise protocol including aerobic and resistance training has been found to improve, the functional capacity is an indirect measure of cardiotoxicity. There is a lack of data in terms of improvement seen in direct measurements of cardiotoxicity such as LVEF and cardiac biomarkers. A lack of evidence regarding the effects of exercise on the direct measurement of cardiotoxicity encourages the need for further research.

## Abbreviations

BNP: B-type natriuretic peptide

CG: Control Group

GLS: global longitudinal strain

HER: human epidermal growth factor receptor

IG: Interventional group

LVEF: left ventricle ejection fraction,

MET: Metabolic equivalent

PRISMA: Preferred Reporting Items for Systematic Reviews and Meta-Analyses

RCT: Randomized control trial

ROS: Reactive oxygen species

RVEF: right ventricular ejection fraction

## Introduction

Globally cancer and cardiovascular diseases cause morbidity and mortality. Despite various advances in cancer treatments, the detrimental effects of these treatment forms cause a significant burden to cancer survivors. The three most prevalent cancers are prostate, colorectal, and melanoma among males, and breast, uterine corpus, and colorectal cancer among females. ^1^ The most common types of childhood cancer are leukemia, brain cancer, lymphoma, and solid tumors. ^2^ Early screening for cancer helps slow down its progress and, if accompanied by appropriate treatment, can result in a significant decline in the mortality rate. ^3^

One of the primary treatment strategies in cancer treatment involves chemotherapeutic drugs such as anthracycline, doxorubicin, Paclitaxel, Cyclophosphamide, and Trastuzumab used alone or in combination with radiation therapy. ^4^^–^^6^ Despite the therapeutic effects of these drugs, cancer survivors have been found to have long-term adverse side effects such as cardiotoxicity, fatigue, cancer-related pain, sleep disorders, and psychological stress, which contribute to morbidity and mortality amongst them. ^7^

Cardiotoxicity is considered a significant concern and severe issue in clinical practice patients receiving chemotherapy. Cardiotoxicity is damage to the heart manifested by either symptomatic or asymptomatic decline in left ventricular ejection fraction (LVEF). ^8^ Although the mechanism of cardiotoxicity is poorly understood, the most agreed upon fact remains the increase of reactive oxygen species (ROS), activating cardiac autophagy and apoptotic pathways. ^9^^–^^12^ Studies have defined cardiotoxicity as a decrease in LVEF of >10% points to a value of <53%(reference value). ^13^ Echocardiography-LVEF, myocardial strain imaging, and cardio biomarkers are the standard marker or parameters for measuring the early and late cardiotoxic effects. ^11^^,^^13^^–^^18^

The proposed strategies to reduce the cardiotoxic effects are: 1) anthracycline dose reduction, 2) altogether avoiding the radiotherapy/blockade of exposure to other areas, 3) usage of iron chelation, 4) treating the preexisting cardiovascular risk factors, 5) using other interventions such as exercise to alleviate its effects.

Exercise training, a most reliable and non-pharmacological option, brings positive outcomes and improves physical fitness by restoring physical function, enhancing the quality of life, and reducing cancer-related fatigue in cancer survivors. This training can be implemented before, during, and after cancer treatments, ^19^^–^^23^ thus nullifying the cardiotoxic effects. Exercise interventions, including aerobic exercises such as treadmill walking, running, cycling, and resistance training such as weight training, strength training using Thera Band, and weight cuffs, are safe and feasible for all cancer populations. Over the past decade, steady growth in the body of evidence supports the importance of exercise in attenuating or mitigating the cardiotoxic effects induced by chemotherapeutic drugs. ^7^^,^^19^^,^^21^^,^^22^^,^^24^^–^^27^ However, several outcome measures, such as VO _2_ max (maximum amount of oxygen utilized during exercise) and metabolic equivalent (MET), are used in measuring cardiotoxicity; the standard direct predictors are LVEF and cardiac biomarkers.

To the best of our knowledge, there is no systematic review on the effects of exercise training on cardiotoxicity in cancer survivors; this review aims to synthesize evidence regarding the role of exercise training on cardiotoxicity and identify potential knowledge gaps in terms of research in this area.

## Methods

This systematic review of clinical trials on the Effects of exercise training on cardiotoxicity in cancer survivors is reported according to the PRISMA guidelines (See Reporting Guidelines). ^28^

### Search strategy and selection criteria

A detailed data search was performed on databases PUBMED, SCOPUS, EMBASE, COCHRANE, and WEB OF SCIENCE from August 2009 to March 2021. The search terms used were cancer, carcinoma, neoplasm (MeSH), cancer survivors (MeSH), adult cancer survivors, and pediatric cancer survivors. For intervention, search terms were exercise training, exercise therapy, prehabilitation, rehabilitation, aerobic training, resistant training, endurance training, treadmill, cycle ergometry, swimming, walking, running, free weights, manual, kettlebell exercises, dumbbell exercises, Pilates, yoga, flexibility training, stretching exercises, high-intensity interval training. For cardiotoxicity outcomes, the search terms are Cardiotoxicity, Cardiopulmonary fitness, functional capacity, left ventricle ejection fraction, heart failure, cardiovascular reserve capacity, coronary vascular disease, and physical fitness. The Boolean operator ‘AND’ or ‘OR ‘combined the search terms. Potentially relevant studies were included from the reference list of the included articles. The search for clinical trials was limited to those involving human participants and those published in English. Two investigators, RR and SRS, independently searched the databases mentioned above. The studies were further screened by RR and SRS based on the preset inclusion criteria. A discussion with VPS sorted any further discrepancies.

### Inclusion criteria

Type of participant: All kinds of cancer survivors who received chemotherapy.

Type of study design: Only clinical trials.

### Exclusion criteria

Studies that use other interventions rather than exercise such as Music therapy and Cognitive behavioral therapy, Nordic Walking, speech therapy qualitative studies, Cross-sectional studies, and Systematic review.

### Data extraction and management

RR and SRS performed full-text screening for the included articles independently, and any disagreements were sorted after discussion with VPS. Information on the objectives, site and cancer stages, adjuvant treatment, intervention details, comparator, outcome measures, study design, sample size, adverse events, and critical findings of the included studies were mentioned in the data extraction sheet.

### Assessment of risk of bias

The included studies underwent the risk of bias assessment performed independently by RR and SRS using the National Heart Lung Blood Institute (NHLBI). ^29^ The NIH checklist for each study type measures 14 unique questions and was scored to assess studies' internal validity. The studies were scored under each query related to randomization, allocation concealment, blinding of participants and assessors, baseline characteristics, dropouts, intervention adherence, outcome data, and other biases. Studies were marked as ‘good,’ ‘fair,’ and ‘poor’ if they met 10-14, 5–9, and ≤4 scores accordingly (See *Underlying data*). ^28^ Discussions with VPS sorted disagreements in the marking system of the studies between the two reviewers.

## Results

### Characteristics of the studies

In this review, 9021 articles were retrieved from a comprehensive search of the following databases; PubMed-611, web of science-1728, Scopus-4058, Embase-1069, Cochrane-1555. A total of 6089 articles were found after merging duplicates. Based on the title and abstract screening, 13 articles were eligible for full-text screening. Out of the 13 articles, seven met the inclusion criteria and were included in this review (See *Underlying data*). ^28^

A quality analysis using NHLBI Questionnaire was performed for the included studies interventional studies. Most of the studies were fair to good in quality, and few studies had missing data, smaller sample sizes, dropouts, and differences in baseline characteristics. Among seven studies, one was a single-arm pre-post intervention design; four were randomized controlled trials (RCTs); two were non-RCT. In all included studies, the patients were diagnosed with breast cancer; however, only few authors reported their stage. Most of the participants included in the studies were those aged above 18. The outcome measure of all included studies was cardiac function using echocardiography- LVEF, Global longitudinal strain (GLS), and circulating cardiac biomarkers (troponins and N terminal-pro brain natriuretic peptide (NT-proBNP). Most of the patients received anthracycline class drugs such as doxorubicin and trastuzumab as adjuvant therapy.

Exercise intervention for the included participants comprised either aerobic or resistance training or a combination of both. Supervised treadmill walking, unsupervised home-based walking, and cycle ergometry were the modalities used in aerobic exercise, and for resistance training, Thera band, dumbbell, and medicine ball were used. Out of seven studies, three studies ^30^^–^^32^ used aerobic, and resistance training as their intervention, and the other four studies ^33^^–^^36^ used only aerobic training for their patients. The duration of these exercises was around 30-60 min performed in about 9-16 weeks. Two out of seven studies ^33^^,^^34^ incorporated exercise bout just 24-hours before the chemotherapy and observed the changes in echocardiographic findings and cardio biomarkers.

The dropouts were as follows: 11 from Foulkes *et al.*, 21 from Katarzyna Hojan *et al.*, two from Howden *et al.*, three from Kirkham *et al.*, two from Haykowsky *et al.,* six from Zhijun Ma *et al.,* and three from Kirkham *et al.*

One study among seven has been published twice in a different journal, and their relevant data has been extracted and presented in Tables 1 and 2.

**Table 1.** Characteristics of the Included Studies.

Author	Cancer site/Stage	Age/Gender	Study design	Sample size	Adjuvant treatment	Exercise intervention	Quality assessment measure
Foulkes *et al*., 2019 ^30^	Breast cancer	female/46-66	non-RCT	28 Usual care; *n* = 14 Exercise; *n* = 14	chemotherapy+radiotherapy	Aerobic and resistance exercise	Fair
Katarzyna Hojan *et al*., 2020 ^31^	Breast cancer IB, IIA, IIB, IIIA	female/18-75 years	RCT	68 Usual care; *n* = 34 Exercise; *n* = 34	trastuzumab therapy +radiotherapy	Endurance and strength exercises	Good
Erin J Howden *et al*., 2019 ^32^	Breast cancer	female/(47-53) ± 9 years	non-RCT	28 Usual care; *n* = 14 Exercise; *n* = 14	anthracycline	Aerobic and resistance exercise	Fair
Amy A. Kirkham *et al*., 2018 ^33^	stage I–III Breast cancer	female/50 ± 9	RCT	24 Usual care; *n* = 11 Exercise; *n* = 13	doxorubicin	Aerobic exercise	Good
Haykowsky *et al*., 2009 ^35^	HER2 positive Breast cancer	53 ± 7 years	single group design (pre-post)	Exercise *; n* = 17	trastuzumab therapy +radiotherapy	Aerobic training	Good
Zhijun Ma *et al.*, 2018 ^36^	Breast cancer	43.1±5 years	RCT	64 Usual care; *n* = 33 Exercise; *n* = 31	anthracycline	Aerobic exercises	Fair
A.A. Kirkham *et al.*, 2017 ^34^	Breast cancer	above 18	RCT	24 Usual care; *n* = 11 Exercise; *n* = 13	doxorubicin	Aerobic exercises	Good

RCT-randomized control trial, HER-human epidermal receptor.

**Table 2.** Intervention characteristics, Outcomes, Result of included studies.

Author	Exercise details		Outcome measure	Result	
Foulkes *et al.* (2019) ^30^	AEROBIC 2 session/week moderate to vigorous intensity for 12 weeks aerobic-cycle ergometer 30 minutes 1 session/week unsupervised 30-60 min home aerobic exercise with moderate intensity	RESISTANCE TRAINING 30 min, moderate to vigorous intensity for 12 weeks	echo- (LVEF; global longitudinal strain), cardio biomarkers (troponin; B-type natriuretic peptide),	Usual LVEF % PRE AC 64.3 ± 5.3 4 Months -3.3 (-0.4, 6.9) 16 Months -6.9 (-11.1, -2.0) BNP, ng/L Pre-AC 39.4 ± 50.0 4 Months -4.1 (-48.2, 39.9) 16 Months -14.5 (-63.8, 34.9) Troponin I, ng/L Pre-AC 2.57 ± 0.79 4 Months 33.43 (1.87, 64.99) 16 Months 2.57 (-0.58, 5.72) GLS, % Pre-AC -19.9 ± 2.3 4 Months 0.9 (-1.4, 1.4) 16 Months 2.4 (-1.2, 3.5)	Exercise Training LVEF % PRE AC 63.8 ± 5.4 4 Months -4.6 (-1.1, 10.3) 16 Months -3.1 (-8.4, 2.1) BNP, ng/L Pre-AC 43.4 ± 29.8 4 Months -0.4 (-30.8, 30.0) 16 Months -10.6 (-50.9, 29.7) Troponin I, ng/L Pre-AC 3.00 ± 1.07 4 Months 20.89 (3.62, 38.12) 16 Months 3.63 (0.50, 6.75) GLS Pre-AC -19.2 ± 1.5 4 Months 0.1 (-1.4, 1.3) 16 Months 0.2 (-1.5, 1.9)
Katarzyna Hojan *et al.* (2020) ^31^	endurance 5 session/week for 9 weeks 2 forms of exercise/one session (walking treadmill, cycling) 45-50 min	strength 5 session/week for 9 weeks isometric, concentric, and eccentric training consisted of one to three sets of 8–10 repetitions	cardiac function-LVEF, RVEF GLS	LVEF (%) Baseline- 63,9 ± 2.72 After- 59.82 ± 4.02 P (0.143) RVEF (%) Baseline 53.3 ± 6.5, After- 54.2 ± 5.2 P (0.488) GLS (%) Baseline 17.3 ± 2.5 After 16.8 ± 2.5	LVEF (%) Baseline 65.69 ± 5.02 After 64.88 ± 5.81 P (0.009) RVEF (%) Baseline 52.8 ± 7.5 After 52.1 ± 6.6 P (0.788) GLS (%) Baseline 17.5 ± 2.5 After 17.6 ± 2.5
Erin J Howden *et al.* (2019) ^32^	AEROBIC 2 session/week for 12 weeks 30 minutes one unsupervised 30–60 minute	RESISTANCE TRAINING 2 session/week for 12 weeks 30 minutes	Echocardiography (LVEF and GLS) cardiacbiomarkers (troponin and B-type natriuretic peptide)	LVEF (%) Pre-treatment 62.8 ± 4.9 post-treatment 59.1 ± 4.1 GLS (%) pre 20.4 ± 2.1 post 19.5 ± 2.0 BNP (ng/L) pre 35.8 ± 39.6 post 36.2 ± 19.7 Troponin I (ng/L) pre-2.6 ± 1.0 post 35.6 ± 27.2	LVEF (%) Pre-treatment 64.1 ± 5.0 post-treatment 60.6 ± 5.4 GLS (%) pre 19.7 ± 2.0 post 19.6 ± 2.0 BNP (ng/L) pre 39.6 ± 31.6 post 40.2 ± 23.4 Troponin I (ng/L) pre-3.2 ± 1.5 post 21.4 ± 16.0
Amy A. Kirkham *et al.* (2018) ^33^	prior to 24 hrs. treatment supervised treadmill 10-min warm-up 30 min vigorous intensity (70% of heart rate reserve (HRR)), and a 5-min cool-down	Nil	Echocardiograms and circulating biomarkers 0–14 days (baseline) and 7–14 days after treatment	Longitudinal strain (%) Baseline − 19.6 ± 1.9 End of T − 20.3 ± 1.6 Cardiac troponin T (pg./mL) Baseline 1.5 ± 2.3 End of T 11.6 ± 8.3 NT-proBNP (pg./mL) Baseline 59 ± 35 End of T 77 ± 39 LVEF (%) Baseline 58 ± 3 After 58 ± 3	Longitudinal strain (%) Baseline − 19.2 ± 1.9 End of T − 18.7 ± 1.4 Cardiac troponin T (pg./mL) Baseline 1.3 ± 2.1 End of T 13.6 ± 11.2 NT-proBNP (pg./mL) Baseline 52 ± 30 End of T 52 ± 30 LVEF (%) Baseline 57 ± 4 After 57 ± 4
Haykowsky *et al.* (2009) ^35^ Pre post study design	3D/week for 4 months (30-60 min) cycle ergometry (60%-90%) peak oxygen consumption	nil	ejection fraction by echocardiography	LVEF (%) Baseline (pre: 64% ± 4%) After 4 months Post: (59% ± 4%)	
Zhijun Ma *et al.* (2018) ^36^	3D/week for 16 weeks 50 min treadmill 60%-70% HR max	nil	ejection fraction by echocardiography serum biomarkers	LVEF (%) Baseline= (pre: 51 ± 5.6 After-post: 47 ± 2.6 NT-pro BNP (ng/L) Baseline-72.1 ± 13.6 After-348.2 ± 25.4	LVEF (%) Baseline: 55 ± 3.5 After: 60 ± 2.9 NT-pro BNP (ng/L) Baseline-84.2 ± 21.5 After-90.6 ± 18.4
A.A. Kirkham *et al.* (2017) ^34^	prior to 24 hrs. treatment supervised treadmill 10-min warm-up, 30 min vigorous intensity (70% of heart rate reserve (HRR)), and a 5-min cool-down	nil	outcome measures were LV longitudinal strain, and cardiac Troponin T (cTnT) Echocardiograms and circulating biomarkers 0–14 days (baseline) and 7–14 days after the last doxorubicin treatment.	Cardiac troponin T (pg./mL) Baseline- 1.5 ± 2.3 after - 1.9 ± 2.6 NT-proBNP (pg./mL) Baseline- 59 ± 35 After- 323 ± 15 LVEF (%) Baseline-58 ± 3 After-59 ± 4 Longitudinal strain (%) Baseline-19.6 ± 1.9 After-21.5 ± 1.6	Cardiac troponin T (pg./mL) Baseline - 1.3 ± 2.1 After- 2.6 ± 3.2 NT-proBNP (pg./mL) Baseline 52 ± 30 After- 214 ± 77 LVEF (%) Baseline-57 ± 4 After-60 ± 3 Longitudinal strain (%) Baseline-19.2 ± 1.9 After-21.4 ± 1.8

RVEF- right ventricular ejection fraction, LVEF –left ventricle ejection fraction, GLS- global longitudinal strain

BNP-B-type natriuretic peptide, AC- Anthracycline class.

## Discussion

Patients with cancer with underlying cardiovascular complications have reduced life expectancy compared to those with cancer alone. It is expected that the survival rate of the cancer population will increase by 30% by 2022 in the United States alone. ^37^ Modern treatment strategies for cancer have improved their survival rate and costed adverse cardiovascular injury as side effects in their long-term survival period. This study aimed to look for therapeutic strategies to alleviate the side effects. ^38^ A growing body of evidence supports the role of exercise in preventing and managing various treatment-related complications in cancer survivors. Hence, this review was conducted to summarize the available literature and thus evaluate the effects of exercise training on cardiotoxicity in cancer survivors. The studies included in this review used outcome measures that directly measure cardiotoxicity in cancer survivors. The effect of exercise interventions has been discussed in detail under each outcome measure.

### Left ventricular ejection fraction

Cancer therapy-induced cardiac dysfunction is a long-term complication in cancer survivors, with some being symptomatic and others asymptomatic. Heart failure is defined as pump failure, measured in LVEF. ^39^ Exercise training potentially induces ventricular remodeling in patients with heart failure ^40^ by restoring abnormal neurohormonal, autonomic and hemodynamic functions. Among the included studies, five studies ^30^^–^^32^^,^^35^^,^^36^ measured LVEF as a primary outcome measure, and the other two studies ^33^^,^^34^ evaluated it as a secondary measure. one ^36^ among seven studies, showed clinically significant improvements in LVEF (P<0.05). In contrast, six other showed negligible changes in left ventricular ejection from baseline to post-chemotherapy. This study incorporated only aerobic exercises for their patients for 16 weeks (3d/week). ^36^

### Global longitudinal strain (GLS)

It is one of the echocardiographic findings and a potential predictor of subclinical cancer therapy-related cardiac dysfunction. It analyzes the subtle changes or deformation occurring in the left ventricle. ^39^^,^^41^ Based on research evidence, a greater than 15% change is a strong predictor of cardiotoxicity. ^41^ There a is lack of evidence supporting the role of exercise training in GLS; however, in a trial conducted by Valzania, Cinzia *et al.,* improvements have been seen in GLS values in patients receiving cardio resynchronization therapy during exercise. ^42^ Among seven studies included in this review, two studies ^35^^,^^36^ didn’t assess GLS as an outcome measure, while most studies showed slight changes in GLS value. However, a clinical trial conducted by Foulkes *et al.* demonstrated a considerable decline in GLS value over 16 months (P = 0.015), ^30^ despite providing a combination of aerobic and resistance training.

### Cardiac biomarkers

Biomarkers are one of the best diagnostic predictors of early cardiotoxicity. The test performed during or after the chemotherapy helps anticipate the presence of cardiotoxicity. Troponins and Natriuretic peptides are the significant biomarkers in determining subclinical cardiotoxicity. These biomarkers imply a certainty of cardiac damage due to chemotherapy. ^39^^,^^43^ Based on the research literature, it is evident that even prolonged exercise training in healthy individuals can cause an acute elevation in these biomarkers, which are transient. ^44^ However, a trial conducted by Braith *et al.* on heart failure patients showed that 16-weeks of endurance training helped reduce the baseline values of natriuretic peptides. ^45^ Only five studies ^30^^,^^32^^–^^34^^,^^36^ investigated biomarkers as their outcome and revealed that there is a significant elevation of troponins and natriuretic peptides post-chemotherapy in acute time. Interestingly these values recovered after 12 months in two studies. ^30^^,^^36^ Thus, exercise training as an intervention to reduce biomarkers level is unclear and poorly understood.

### Strength and limitation

Thus, this systematic review summarizes the effect of exercise training on cardiotoxicity in cancer survivors. Although previous studies summarize the impact of exercise as an intervention on cardiotoxicity measured by VO _2_ max in cancer survivors, no review synthesizes evidence regarding the direct measure of cardiotoxicity. The inclusion of studies published in English and exclusion of the grey literature are the limitation of this review. Aerobic exercise training was limited to treadmill modality in most of the studies. Recent advances in exercise training like high-intensity interval training have shown clinical benefits in reduced ejection fraction patients suggesting ventricular remodeling, thus improving their functional capacity. ^46^ So, there is a need for an alternate form of exercise to counteract the chemotherapy-induced dysfunction.

In this review, one out of seven studies ^36^ showed statistically significant improvement in cardiotoxicity-related outcome measures. In comparison, three studies ^30^^,^^32^^,^^33^ showed that there was no deterioration of the cardiotoxicity related outcome measures. In contrast, the remaining three studies ^30^^,^^32^^,^^35^ showed a decline in the outcome measures, which was statistically significant.

Individualized exercise prescription, based on the frequency, intensity, type, and time (FITT) principle, can be recommended based on patients’ baseline characteristics or comorbidities limiting their physical performance. Cancer patients also suffer from sarcopenia, ^47^^–^^49^ which reduces their strength; there is a clinical need for resistance training.

The timing of exercise intervention used before, concomitant, or after chemotherapy has a significant role in providing protective effects. A gap has to be explored for further strengthening of evidence in optimal timing. In most of the included studies, the intervention duration was short, about four months; other studies can implement exercises for the long run and see the clinical changes.

Childhood cancer survivors with Hodgin’s lymphoma, and adult cancer survivors with prostate and colorectal cancer, are common and may also suffer from chemotherapy-related side effects. Predominantly early childhood cancer survivors with a longer life span have to sustain chemotherapy-induced cardiovascular injury affecting their quality of life in the long run. Despite evidence regarding exercise training, no studies are measuring the clinical changes in LVEF, GLS, and biomarkers, which are the direct measures of cardiotoxicity. Therefore, exploring the role of exercises in other cancer survivors is crucial to gather more evidence regarding the direct measurement of cardiotoxicity.

## Conclusion

This review concludes that exercise has a potential role as an intervention in preventing deterioration of outcome measures that measure cardiotoxicity and improve the same. We recommend further research to ascertain the dose, volume of exercise, and optimal timing to further understand the role of exercise in cardiotoxicity.

## Data availability

### Underlying data

Open Science Framework (OSF): Effects of exercise on cardiotoxicity in cancer survivors. A systematic review’. DOI: 10.17605/OSF.IO/Q4YZM

This project contains the following underlying data:

Review protocol.docx. (It has information on search strategy, databases, search terms used, and inclusion and exclusion criteria.)

Systematic review.xlsx. (Patients’ characteristics such as site/stage, adjuvant treatments, intervention, results, dropouts)

Risk of bias.docx. (It includes the tables for which risk of bias for studies was done using NIH tool)

Data are available under the Creative Commons Attribution 4.0 International license ( CC-BY 4.0).

## Reporting guidelines

Open Science Framework (OSF): PRISMA checklist and flow chart for ‘Effects of exercise training on cardiotoxicity in cancer survivors. A systematic review’. DOI: 10.17605/OSF.IO/Q4YZM

Data are available under the terms of the Creative Commons Attribution 4.0 International license ( CC-BY 4.0).

## Author contributions

Conceptualization, R.R. AND S.R.S; Methodology, R.R. Investigation, R. R AND S.R.S.

Software, R.R. AND S.R.S; Supervision, S.R.S., V.P.S. AND S.B.; Funding Acquisition, S.R.S., V.P.S. AND S.B.; Writing – Original Draft Preparation, Writing – Review & Editing, R. R. AND S.R.S.
