# Supplementary material for: Effects of exercise training on cardiotoxicity in cancer survivors. A systematic review
Source: F1000Res. 2026 Jul 27;11:497. Originally published 2022 May 5. [Version 2] doi: 10.12688/f1000research.112667.2 (PMC13641239; doi:10.12688/f1000research.112667.2)
Supplement: Peer review history

# Reviewer report: Reviewer response for version 2

**DOI:** 10.5256/f1000research.199218.r505310

**Published:** 2026-08-18

**Authors:** Sheetal Kalra

**Affiliations:** 1 School of Physiotherapy, Delhi Pharmaceutical Sciences and Research University, New Delhi, Delhi, India

All the changes and revisions suggested previously have been incorporated by the authors in the revised manuscript.

Are the rationale for, and objectives of, the Systematic Review clearly stated?

Yes

Is the statistical analysis and its interpretation appropriate?

Yes

Are sufficient details of the methods and analysis provided to allow replication by others?

Yes

Are the conclusions drawn adequately supported by the results presented in the review?

Yes

Reviewer Expertise:

Physical health, fitness, sports injuries, sports rehabilitation, women health, exercise interventions

I confirm that I have read this submission and believe that I have an appropriate level of expertise to confirm that it is of an acceptable scientific standard.

# Reviewer report: Reviewer response for version 1

**DOI:** 10.5256/f1000research.124396.r168414

**Published:** 2024-09-11

**Authors:** Sheetal Kalra

**Affiliations:** 1 School of Physiotherapy, Delhi Pharmaceutical Sciences and Research University, New Delhi, Delhi, India

This is an interesting article. The authors have synthesized the evidence regarding role of exercises on cardiotoxicity.

 Certain observations are:

 1. In Abstract it would be justified to include a line or rationale of study , may be in background or as separate heading

 2. Methodology section can be improved by including information under PICOS framework

 3. The PRISMA flowchart should include reasons for exclusion of 6076 articles in screening section.

 4. Also records identified does not give complete information. Should be re-written.

 5. How was the review Question1 "What are the effects of exercise training in preventing cardiotoxicity in cancer survivors" mentioned in study protocol addressed in study?

Are the rationale for, and objectives of, the Systematic Review clearly stated?

Yes

Is the statistical analysis and its interpretation appropriate?

Yes

Are sufficient details of the methods and analysis provided to allow replication by others?

Yes

Are the conclusions drawn adequately supported by the results presented in the review?

Yes

Reviewer Expertise:

Physical health, fitness, sports injuries, sports rehabilitation, women health, exercise interventions

I confirm that I have read this submission and believe that I have an appropriate level of expertise to confirm that it is of an acceptable scientific standard, however I have significant reservations, as outlined above.

# Response

**Published:** 2026-04-22

**Authors:** Stephen Samuel

**Affiliations:** Manipal Academy of Higher Education, India

1. In Abstract it would be justified to include a line or rationale of study , may be in the background or as separate heading

**Reply: **Dear reviewer, thanks for your comment. I have made necessary changes in the abstract.

 2.Methodology section can be improved by including information under PICOS framework..

**Reply:**Dear reviewer, thanks for your comment.  On basis of **PIO** framework, the inclusion criteria has been framed.

 3. The PRISMA flowchart should include reasons for exclusion of 6076 articles in the screening section

**Reply: **Dear reviewer, thanks for your comment. I have added the necessary changes in the Prisma flowchart.

 4. Also, the records identified does not give complete information. Should be re-written

**Reply: **Dear reviewer, thanks for your comment. I have added the necessary changes in the records identified section -giving no of articles from each database has been updated

 5. How was the review Question1 "What are the effects of exercise training in preventing cardiotoxicity in cancer survivors" mentioned in study protocol addressed in the study?

**Reply: **Dear reviewer, thanks for your comment.

 Regarding the question, the role of exercise training in preventing cardiotoxicity, our study didn’t find evidence for that.

 The conclusion is modified and updated with evidence found. Stated  exercise improves functional capacity  and neither prevents or mitigates the cardiotoxicity, since the quality of evidence is inconsistent. Needed a large studies with RCT designs.

# Reviewer report: Reviewer response for version 1

**DOI:** 10.5256/f1000research.124396.r180450

**Published:** 2023-07-12

**Authors:** Quentin Jacquinot

**Affiliations:** 1 University of Franche-Comté, Besançon, France

**Abbreviations section (page 3)**

- Harmonize the writing. For example: Capitalize the first only or capitalize first letter for each word.
- Write the meaning of MET in full. Add "of Task".

**Introduction section (page 3)**

-  Add a reference for the first sentence.
- Change the order of the sentences in the writing of the first paragraphs of the introduction for more logic. For example: Start with the incidence (ref 1 and 2), then the notion of mortality (ref 3) then the sentences of references 4-6 and finally the first two sentences of the introduction and the reference.
- Weight your comments regarding the sentence "This training can be implemented before, during, and after cancer treatments,19–23 thus nullifying the cardiotoxic effects". Modify "can be" by "could be" and "nullifying" by a less "strong" term because it contradicts the results of your article.
- Regarding VO _2_, specify that it is an "indirect" reflection of cardiotoxicity.

**Methods section**

- Cite reference 28 (See Underlying data) earlier and in each subsections of the method section because currently it comes too late, whereas these informations are important.

***subsection " Search strategy and selection criteria"***

- Add the words MESH for all categories (intervention, cardiotoxicity, etc...) as done for neoplasm and cancer survivors.

***subsection "Inclusion criteria"***

- Specify the type of study design (RCT, observational, etc.)

***subsection "Exclusion criteria"***

- Specify that pre-clinical studies are excluded.
- Why Nordic walking is excluded whereas selected studies offered unsupervised walking or the treadmill (cf : Supervised treadmill walking, unsupervised home-based walking in results section)?

**Results section**

***Subsection "Characteristics of the studies"***

- Maybe add the flowchart in the body text and not in the supplementary data?
- In the first sentence put the number in brackets like this: PubMed (n=6).
- Add "®" to Thera band® as it's a brand or change by "resistance bands".
- In table 2: Write in the "results" column "usual" and "training" at the top and not only in the first line. This will make it possible to harmonize the wording to facilitate reading on the 2 pages.
- Same comment: Harmonize the wording of the "outcomes measures" column because sometimes it writes echo* - sometimes echography in full then sometimes no information on the method (as for Katarzyna et al).
- Same comment for the "outcomes measure" column: Sometimes there are deadlines, sometimes not. You must harmonize the wording and be more rigorous in the wording and organization of the paragraphs so that they are written in the same way for all the studies.
- Last sentence of the result part, please add the reference of the study.

**Discussion section**

- First sentence of the discussion part, please add a reference.
- Specify within the limits that the data presented relates only to breast cancer.

Are the rationale for, and objectives of, the Systematic Review clearly stated?

Yes

Is the statistical analysis and its interpretation appropriate?

Not applicable

Are sufficient details of the methods and analysis provided to allow replication by others?

Yes

Are the conclusions drawn adequately supported by the results presented in the review?

Yes

Reviewer Expertise:

Exercise physiology and supportive care

I confirm that I have read this submission and believe that I have an appropriate level of expertise to confirm that it is of an acceptable scientific standard, however I have significant reservations, as outlined above.

# Response

**Published:** 2026-04-22

**Authors:** Stephen Samuel

**Affiliations:** Manipal Academy of Higher Education, India

- Harmonize the writing. For example: Capitalize the first only or capitalize first letter for each word.
- Write the meaning of MET in full. Add "of Task".

**Reply:** Dear reviewer, thank you for your comment. The changes have been made.(Abbreviations are corrected.

**Introduction section (page 3)**

-  Add a reference for the first sentence.
- Change the order of the sentences in the writing of the first paragraphs of the introduction for more logic. For example: Start with the incidence (ref 1 and 2), then the notion of mortality (ref 3) then the sentences of references 4-6 and finally the first two sentences of the introduction and the reference.
- Weight your comments regarding the sentence "This training can be implemented before, during, and after cancer treatments,19–23 thus nullifying the cardiotoxic effects". Modify "can be" by "could be" and "nullifying" by a less "strong" term because it contradicts the results of your article.
- Regarding VO _2_, specify that it is an "indirect" reflection of cardiotoxicity.

**Response: **Dear reviewer, thank you for your comment, the necessary changes have been made in the introduction.

**Methods section**

- Cite reference 28 (See Underlying data) earlier and in each subsections of the method section because currently it comes too late, whereas these informations are important.

**Response: Dear** reviewer, thank you for your comment, the reference section is updated with the necessary changes.

***subsection " Search strategy and selection criteria"***

- Add the words MESH for all categories (intervention, cardiotoxicity, etc...) as done for neoplasm and cancer survivors.

**Response: Dear** reviewer, thank you for your comment. The mesh terms are updated.

***subsection "Inclusion criteria"***

- Specify the type of study design (RCT, observational, etc.)

**Response: Dear** reviewer, thank you for your comment(inclusion criteria is refined and type of study design are added )

***subsection "Exclusion criteria"***

- Specify that pre-clinical studies are excluded.
- Why Nordic walking is excluded whereas selected studies offered unsupervised walking or the treadmill (cf : Supervised treadmill walking, unsupervised home-based walking in results section)?

**Response: Dear** reviewer, thank you for your comment. The necessary changes have been made.  We acknowledge that it could be a part of interventions, but Nordic walking requires skilled walking and the use of a pole, which has more heterogeneity.

**Results section**

***Subsection "Characteristics of the studies"***

- Maybe add the flowchart in the body text and not in the supplementary data?
- In the first sentence put the number in brackets like this: PubMed (n=6).
- Add "®" to Thera band® as it's a brand or change by "resistance bands".
- In table 2: Write in the "results" column "usual" and "training" at the top and not only in the first line. This will make it possible to harmonize the wording to facilitate reading on the 2 pages.
- Same comment: Harmonize the wording of the "outcomes measures" column because sometimes it writes echo* - sometimes echography in full then sometimes no information on the method (as for Katarzyna et al).
- Same comment for the "outcomes measure" column: Sometimes there are deadlines, sometimes not. You must harmonize the wording and be more rigorous in the wording and organization of the paragraphs so that they are written in the same way for all the studies.
- Last sentence of the result part, please add the reference of the study.

**Response: Dear** reviewer, the flow chart was added in the body text at first ,but as per the journal instruction its been added as supplementary material.

**Response: **Dear reviewer in Table 2 , the necessary changes have been made in outcome measures column.

**Response: **Dear reviewer, the reference has been updated to the last sentence of the results part.

**Discussion section**

- First sentence of the discussion part, please add a reference.
- Specify within the limits that the data presented relates only to breast cancer.

**Response: **Dear reviewer, thanks for your comment. The first sentence of the discussion part is referenced with 37 and updated.

**Response: **Dear reviewer, thanks for your comment. I have added the necessary changes to the discussion part( limited to breast cancer).

# Reviewer report: Reviewer response for version 1

**DOI:** 10.5256/f1000research.124396.r140895

**Published:** 2022-07-01

**Authors:** Sharon F. Kramer

**Affiliations:** 1 Faculty of Health, School of Nursing and Midwifery, Deakin University, Geelong, Vic, Australia

Thank you for the opportunity to review this manuscript of a systematic review investigating the effect of exercise on cardiotoxicity outcomes in cancer patients receiving chemotherapy and/or radiotherapy.

 The rationale for the review is clearly stated. The background is missing some details regarding definitions of the main concepts addressed in this review for example: cardiotoxicity and details about how this is measured (this information is reported in the discussion and could be moved to the background and methods) and exercise and what is the potential underlying mechanisms of exercise on cardiotoxicity.

 Methods:

- Several sections are missing in the methods.
- What criteria are used regarding study design? i.e., randomised controlled trail, non-randomised controlled trials, pre-post studies?
- What was the process of screening title and abstract?
- What comparisons were of interest? i.e., exercise A vs exercise B, exercise vs no exercise etc.
- I suggest providing more background about why these tools were developed as I was not aware of these tools and maybe other readers might not be either. For example, it is not clear that there are several tools that were developed for different study types. Specify which tools were used.
- Add a section to the methods about the outcomes of interest including: timing of outcome measurement of interest (post intervention and FU), how these outcomes are/should be measures, and how the outcome measure should be interpreted higher value is greater toxicity? (some of the details are reported in discussion and could be moved to methods section).
- Although no meta-analyses were performed, the methods still need a section about how the data was summarised/synthesised. Please provide information abut how the results were structured by study design, by population, by outcome.

Results:

- The main results regarding toxicity are reported in the discussion and should be moved to the results section. I suggest restructuring or adding a table with just the results outcomes and consider how this could be structured (see suggestions above by study design, outcome or population).
- Avoid vote counting i.e. 1 out of seven studies showed a significant result. Reporting 95%CI is more informative and helps the reader to interpret data better instead of reporting just the p -value.

Conclusion:

- The conclusion currently doesn’t reflect what is reported in the results. Currently there doesn’t seem to be any strong evidence to suggest that exercise mitigates the cardiotoxic effects of chemo/radiotherapy in cancer patients.

I suggest revisiting the PRISMA guidelines to help with structuring the manuscript and adding missing information/sections.

Are the rationale for, and objectives of, the Systematic Review clearly stated?

Yes

Is the statistical analysis and its interpretation appropriate?

No

Are sufficient details of the methods and analysis provided to allow replication by others?

No

Are the conclusions drawn adequately supported by the results presented in the review?

No

Reviewer Expertise:

Systematic review methods including meta-analyses and data synthesis, risk of bias assessment; clinical trials and exercise

I confirm that I have read this submission and believe that I have an appropriate level of expertise to confirm that it is of an acceptable scientific standard, however I have significant reservations, as outlined above.

# Response

**Published:** 2026-04-22

**Authors:** Stephen Samuel

**Affiliations:** Manipal Academy of Higher Education, India

1)The rationale for the review is clearly stated. The background is missing some details regarding definitions of the main concepts addressed in this review, for example cardiotoxicity and details about how this is measured (this information is reported in the discussion and could be moved to the background and methods) and exercise and what is the potential underlying mechanisms of exercise on cardiotoxicity.

**Response:** Dear reviewer, Thank you for your comments, the description about cardiotoxicity and its measures was already mentioned in the introduction in 6 ^th^ line of 3 ^rd^ paragraph. . We have also updated the mechanism of how exercise works in the 9 ^th^ line of the 5th paragraph.

 2.Methods:

- Several sections are missing in the methods.

**Response: **Dear reviewer, thank you for your comments. We have made significant changes in the methods section.

- What criteria are used regarding study design? i.e., randomized controlled trail, non-randomised controlled trials, pre-post studies?

**Response: **Dear reviewer, thanks for your comments. We have added the criteria and it is updated in the method section under inclusion criteria.

 What was the process of screening title and abstract?

 Dear reviewer, thanks for your comments. We have added the screening details in the method section.

              

- What comparisons were of interest? i.e., exercise A vs exercise B, exercise vs no exercise, etc.

**Response: **Dear reviewer, thanks for your comments. We are focused on how exercise can help the risk of cardiotoxicity;so any kind of exercise is considered, the comparison was not in our scope of review, as there is lot of variation in study designs. we are unable to do comparison. 

- I suggest providing more background about why these tools were developed as I was not aware of these tools and maybe other readers might not be either. For example, it is not clear that there are several tools that were developed for different study types. Specify which tools were used.

**Response:** Dear reviewer, the NHLBI(national heart lung and blood institute) has its different study tools for varied study designs. The details are available and updated in the risk of bias section.

- Add a section to the methods about the outcomes of interest including: timing of outcome measurement of interest (post intervention and FU), how these outcomes are/should be measures, and how the outcome measure should be interpreted higher value is greater toxicity? (Some of the details are reported in discussion and could be moved to methods section).

**Response:**Dear reviewer, thank you for your comments. The measures of cardiotoxicity (outcomes) have been already explained in the introduction. Regarding the interpretation, which is greater toxicity, there is no standardized set value for this. Only if there is critical changes seen in cardiotoxicity outcomes (LVEF, GLS, cardiac biomarkers) from baseline to follow up time period, the studies have considered toxicity.

  

- Although no meta-analyses were performed, the methods still need a section about how the data was summarized/synthesized. Please provide information about how the results were structured by study design, by population, by outcome.                    

**Response: **Dear reviewer, thank you for your comment. The data of the included articles were summarised and added to the Excel sheet. This information has been mentioned under the data extraction heading of the method section

 3. Results

- The main results regarding toxicity are reported in the discussion and should be moved to the results section. I suggest restructuring or adding a table with just the results outcomes and consider how this could be structured (see suggestions above by study design, outcome or population).

**Response: **Dear reviewer, thank you for your comment. We have mentioned the main results considering the cardiotoxicity in the result section under the 3 ^rd^ paragraph.

 All the articles retrieved and the data of the included studies is reported in the table under the results section. So the discussion part is more focused on exercise effects and cardiotoxicity outcomes.
